# Joint Trajectories of Multimorbidity, Function, Cognition, and Depression in the HRS (1998-2016)

**DOI:** 10.1093/geroni/igab046.2345

**Published:** 2021-12-17

**Authors:** Corey Nagel, Heather Allore, Jason Newsom, Anda Botoseneanu, David Dorr, Stephen Thielke, Jeffrey Kaye, Ana Quiñones

**Affiliations:** 1 University of Arkansas for Medical Sciences, Little Rock, Arkansas, United States; 2 Yale School of Medicine, New Haven, Connecticut, United States; 3 Portland State University, Portland, Oregon, United States; 4 University of Michigan - Dearborn, Dearborn, Michigan, United States; 5 Oregon Health & Science University, Portland, Oregon, United States; 6 University of Washington, University of Washington, Washington, United States

## Abstract

There is substantial heterogeneity in the impact of multimorbidity on functional, cognitive, and emotional health. Few studies, however, have examined this simultaneously across these multiple domains. We used finite mixture modeling to identify latent clusters of individuals following similar joint trajectories of multimorbidity, functional ability, cognitive performance, and depressive symptoms among 11,841 HRS respondents aged 65+ from 1998 to 2014. We identified four distinct clusters of joint trajectories: (1) 32.5% of the sample were characterized by low multimorbidity (mean = 0.60 conditions at age 65; 2.2 conditions at age 90), minimal deterioration in functional or cognitive ability, and low depressive symptoms; (2) 33.5%, had increased multimorbidity compared with the first group (mean = 2.3 at age 65; 4.0 at age 90) but minimal functional or cognitive impairment and low depressive symptoms; (3) 19.9%, had relatively low multimorbidity (mean = 1.3 at age 65 increasing to 2.8 at age 90), but exhibited worsening cognitive performance, increasing functional limitations, and moderate depressive symptoms ; and (4) 14.1%, had higher multimorbidity (mean = 3.3 at age 65 increasing to 4.6 at age 90), worsening cognitive performance, substantial functional limitation, and high depressive symptoms. Black and Hispanic race/ethnicity, lower levels of income and education, male gender, and smoking history were significantly associated with membership in classes characterized by higher multimorbidity, cognitive and functional impairment, and greater depressive symptoms. This study provides insight into the heterogenous trajectories of aging and helps identify older individuals at higher risk for poor aging outcomes across multiple health domains.

